# Modern Approaches for the Treatment of Heart Failure: Recent Advances and Future Perspectives

**DOI:** 10.3390/pharmaceutics14091964

**Published:** 2022-09-17

**Authors:** Irene Paula Popa, Mihai Ștefan Cristian Haba, Minela Aida Mărănducă, Daniela Maria Tănase, Dragomir N. Șerban, Lăcrămioara Ionela Șerban, Radu Iliescu, Ionuț Tudorancea

**Affiliations:** 1 Cardiology Clinic, “St. Spiridon” County Clinical Emergency Hospital, 700111 Iași, Romania; 2 Department of Internal Medicine, “Grigore T. Popa” University of Medicine and Pharmacy, 700115 Iași, Romania; 3 Department of Physiology, “Grigore T. Popa” University of Medicine and Pharmacy, 700115 Iași, Romania; 4 Internal Medicine Clinic, “St. Spiridon” County Clinical Emergency Hospital, 700115 Iași, Romania; 5 Department of Pharmacology, “Grigore T. Popa” University of Medicine and Pharmacy, 700115 Iași, Romania

**Keywords:** heart failure, angiotensin receptor–neprilysin inhibitor, sodium-glucose co-transporter-2 inhibitors, soluble guanylate cyclase activator, cardiac myosin activation, autonomic modulation

## Abstract

Heart failure (HF) is a progressively deteriorating medical condition that significantly reduces both the patients’ life expectancy and quality of life. Even though real progress was made in the past decades in the discovery of novel pharmacological treatments for HF, the prevention of premature deaths has only been marginally alleviated. Despite the availability of a plethora of pharmaceutical approaches, proper management of HF is still challenging. Thus, a myriad of experimental and clinical studies focusing on the discovery of new and provocative underlying mechanisms of HF physiopathology pave the way for the development of novel HF therapeutic approaches. Furthermore, recent technological advances made possible the development of various interventional techniques and device-based approaches for the treatment of HF. Since many of these modern approaches interfere with various well-known pathological mechanisms in HF, they have a real ability to complement and or increase the efficiency of existing medications and thus improve the prognosis and survival rate of HF patients. Their promising and encouraging results reported to date compel the extension of heart failure treatment beyond the classical view. The aim of this review was to summarize modern approaches, new perspectives, and future directions for the treatment of HF.

## 1. Introduction

Heart failure (HF) is a progressively deteriorating medical condition that is associated with a high risk of hospitalization and unscheduled hospital visits and significantly reduces the patients’ life expectancy and quality of life [1]. Although epidemiological studies report that heart failure affects about 1 to 2% of the general adult population, the true prevalence of HF is likely closer to 4%, as it may be frequently undiagnosed or misdiagnosed, as in the case of heart failure with preserved ejection fraction (HFpEF). Thus, HF is a major public health issue, as well as a significant and ever-increasing socioeconomic burden [2]. The number of patients living with HF is continuously increasing due to a plethora of factors such as ageing population; improved survival following cardiac events such as myocardial infarction; and because of a rising incidence of comorbidities such as hypertension, atrial fibrillation, and type 2 diabetes [3].

Over the last 30 years, the medical management of HF has significantly progressed, thus leading to the amelioration of quality of life and outcomes, especially for patients with reduced left ventricular ejection fraction (HFrEF). The discovery of various pathological mechanisms has made this possible and it has led to a better comprehension of heart failure and thus to the development of novel and effective therapies. Despite recent advances in the pathophysiology of HF and the breakthroughs in the pharmacological and non-pharmacological management of chronic HF, the overall patients’ prognosis remains poor. Thus, the research and discovery of new underlying mechanisms of HF physiopathology pave the way for the development of novel HF therapeutic approaches [4]. In this review, we aimed to summarize the current pharmacological and non-pharmacological strategies, and also we highlighted the new perspectives and future directions regarding HF treatment.

## 2. Pharmacological Therapies for Heart Failure

### 2.1. Pharmacological Therapies for HF: Angiotensin Receptor–Neprilysin Inhibitor (ARNI)

The autonomic nervous system, the renin–angiotensin–aldosterone system (RAAS), and the natriuretic peptide (NP) system play a pivotal role in the modulation of the mechanisms involved in the development and progression of HF [5]. It is well established that the RAAS overactivation in patients with HF leads to increased aldosterone levels and sympathetic tone, vasoconstriction, high levels of arterial blood pressure, and pathological cardiac remodelling [5]. NPs induce various beneficial effects such as natriuresis, vasodilation, antiproliferative properties, vascular remodeling, and a benefic modulation of RAAS. Therefore, growing evidence indicates a myriad of positive outcomes of NPs for the treatment of HF. Additionally, experimental studies have confirmed that neprilysin (a membrane-bound endopeptidase)-induced NP degradation will mitigate all the above-mentioned beneficial effects. Accordingly, the inhibition of neprilysin to increase the plasma concentration of NPs became a promising approach for the treatment of HF [6]. Unfortunately, the inhibition of the neprilysin alone resulted in elevated angiotensin II plasma levels, thus counteracting the vasodilatory effects of neprilysin [7]. To overcome this drawback, angiotensin receptor blockers were combined with neprilysin inhibitors, ushering in the notion of angiotensin receptor–neprilysin inhibitor (ARNI) [7].

Omapatrilat was the first drug developed for the inhibition of both ACE and neprilysin pathways, but the results from the OVERTURE (Omapatrilat Versus Enalapril Randomized Trial of Utility in Reducing Events) trial did not show superior benefits when compared to angiotensin-converting enzyme inhibitor (ACEi) alone in lowering heart failure hospitalization rate or mortality risk [7]. Due to an increased incidence of angioedema induced by omapatrilat reported by the OCTAVE (Omapatrilat Cardiovascular Treatment Assessment Versus Enalapril) study, further development of this medication was discontinued [8]. The PARADIGM-HF (Prospective Comparison of ARNI With ACEI to Determine Impact on Global Mortality and Morbidity in Heart Failure) trial showed promising results since the combination sacubitril–valsartan was superior to enalapril alone in decreasing the risk of death from cardiovascular causes, all-cause mortality, hospitalization for heart failure (HHF) and HF symptoms, and physical limitations [9].

At the time of enrollment, all patients in the PARADIGM-HF trial were hemodynamically stable and treated with an ACEi or angiotensin receptor blocker (ARB). The goal of the PIONEER-HF (Comparison of Sacubitril–Valsartan versus Enalapril on Effect on NT-proBNP in Patients Stabilized From an Acute Heart Failure Episode) study was to assess the safety and effectiveness of sacubitril–valsartan in comparison with enalapril in hospitalized patients with worsening HF, more than half of whom were not receiving neither an ACEi nor an ARB at the point of enrollment [10]. Surprisingly, the introduction of sacubitril–valsartan in the treatment of patients with HFrEF hospitalized for acute decompensated HF lowered N-terminal pro-B-type natriuretic peptide (NT-proBNP) levels more than enalapril alone. Moreover, the patients treated with sacubitril–valsartan showed comparable rates of worsening renal function, hyperkalemia, symptomatic hypotension, and angioedema when compared to those treated with enalapril [10]. The findings of the PIONEER-HF trial extended the indication of sacubitril–valsartan to patients hospitalized for acute decompensated HF, patients with newly diagnosed HF, and patients without previous conventional therapy with RAAS inhibitors [11]. Intriguingly, in the PARAGON-HF (Angiotensin–Neprilysin Inhibition in Heart Failure with Preserved Ejection Fraction) trial in which sacubitril–valsartan was compared to valsartan alone but in patients with HFpEF, the primary composite outcome of total HF and death from cardiovascular causes did not vary substantially between the two groups [12].

Taken together, all these data indicate that ARNI is a promising approach for the treatment of HF. Thus, in the American College of Cardiology Foundation (ACCF), American Heart Association (AHA), and Heart Failure Society of America (HFSA) guidelines, ARNI therapy became a class 1A recommendation, and it should be the primary renin–angiotensin modulator, whereas ACEi or ARB may be used if ARNI therapy is not possible [13]. Meanwhile, the latest European Society of Cardiology (ESC) guidelines give ARNI therapy a 1B recommendation class, indicating that it may be used as an alternative for ACEi in symptomatic HFrEF patients despite optimal medical therapy (OMT) to reduce the risk of HF hospitalization and death [14].

### 2.2. Pharmacological Therapies for HF: Sodium-Glucose Co-Transporter-2 Inhibitors (SGLT2i)

While sodium-glucose co-transporter-2 inhibitors (SGLT2i) were first developed as oral drugs to lower blood glucose by the inhibition of renal tubular sodium-glucose cotransporters, large randomized controlled studies have recently shown that SGLT2i improve cardiovascular outcomes independent of diabetes, along with reducing the risk of HF hospitalization, cardiovascular death, and all-cause mortality [15,16,17,18]. Although the mechanisms of action of SGLT2i to improve outcomes in HF are not fully understood, various hypotheses have been postulated, such as improvements in myocardial energetics and loading conditions, beneficial effects on endothelial function and inflammation, and a delay in the progression of kidney disease [19,20,21]. Taken together, these actions may explain the early and persistent improvements in filling pressures and ventricular remodeling, thus leading to the improvement of cardiovascular outcomes in HF patients [22,23,24].

In the EMPA-REG OUTCOME (Empagliflozin Cardiovascular Outcome Event Trial in Type 2 Diabetes Mellitus Patients–Removing Excess Glucose), the first trial that assessed the impact of SGLT2i on cardiovascular outcomes in patients with type 2 diabetes mellitus (T2DM), empagliflozin showed a lower rate of the primary composite outcome of cardiovascular death, and also a reduced incidence of any cause of mortality or HF hospitalization versus placebo [25]. Interestingly, the cardiovascular effects were independent of renal function and glucose levels [26]. In the CANVAS (Canagliflozin Cardiovascular Assessment Study) and CANVAS-R (Canagliflozin on Renal Endpoints in Adult Participants with Type 2 Diabetes Mellitus) trials, canagliflozin showed cardiovascular benefits since it had a lower risk of cardiovascular events and a significantly reduced exploratory endpoint of HF hospitalization [27]. In the DECLARE-TIMI 58 (Dapagliflozin Effect on Cardiovascular Events–Thrombolysis in Myocardial Infarction 58) interventional clinical trial, dapagliflozin was shown to be superior to the placebo in improving glycemic control, in reducing the relative risk of major adverse cardiac events by 16% among patients with prior myocardial infarction, and in lowering HF hospitalization and cardiovascular and all-cause mortality in patients with HFrEF [28]. The results of these three cardiovascular outcome trials (CVOT) have been confirmed by real-world studies, including CVD-REAL (Comparative Effectiveness of Cardiovascular Outcomes in New Users of SGLT-2 Inhibitors) and the ongoing EMPRISE (Empagliflozin Comparative Effectiveness and Safety Retrospective Study) [29,30].

The remarkable amount of data demonstrating the beneficial effects of SGLT2i has prompted more studies into their potential implications for cardiovascular events and mortality in broader cohorts, which are not confined to diabetes groups. On that account, an increasing number of studies, including two major randomized controlled trials, DAPA-HF (Effect of Dapagliflozin on the Incidence of Worsening Heart Failure or Cardiovascular Death in Patients with Chronic Heart Failure) and EMPEROR-Reduced (Empagliflozin Outcome Trial in Patients with Chronic Heart Failure with Reduced Ejection Fraction), examined the effects of SGLT2i in both diabetic and non-diabetic HF patients [31,32]. Almost 5000 patients with HF New York Heart Association (NYHA) class II to IV and an ejection fraction (EF) < 40% were included in the DAPA-HF trial [31]. The patients treated with SGLT2i versus the placebo group showed a significantly reduced risk of the primary composite outcome of worsening heart failure (hospitalization/urgent visit leading to intravenous therapy for HF) or death from cardiovascular causes [31]. Similar outcomes were also achieved with empagliflozin in the EMPEROR-Reduced trial [33]. Remarkably, in both of these clinical trials, SGLT2i had similar effects in patients with or without T2DM, suggesting that this class of medication has beneficial effects on HF, irrespective of its anti-diabetic actions [31,32,33,34]. Taken together, all these encouraging results from both experimental and clinical studies have led to the introduction of SGLT2i into the current clinical guidelines as a Class 1A recommendation for the treatment of HFrEF [35,36].

Approximately half of all HF patients suffer from HFpEF, and it is expected that this group of HF patients will increase due to a prolonged life expectancy and a growing prevalence of comorbidities (i.e., hypertension, diabetes, obesity) that are now recognized as direct contributors to HFpEF [37]. Unlike HFrEF, in which a plethora of drugs such as beta-blockers, RAAS inhibitors, or SGLT2i are available, in HFpEF, a lack of proven efficient therapy still exists. Thus, studies to determine if SGLT2i might be beneficial in patients with HFpEF were also conducted. The PRESERVED-HF (Dapagliflozin in PRESERVED Ejection Fraction Heart Failure) interventional clinical trial evaluated the hypothesis that dapagliflozin treatment would improve symptoms, physical limits, and exercise capacity in HFpEF patients [38]. Surprisingly, after 12 weeks of dapagliflozin treatment, a significant and consistent clinical improvement was achieved across all predefined subgroups, including patients with and without T2DM and those with EF above and below 60% [38]. The primary outcome of the DELIVER (Dapagliflozin Evaluation to Improve the Lives of Patients with Preserved Ejection Fraction Heart Failure) interventional trial was to assess whether dapagliflozin would reduce cardiovascular death, HF hospitalization, or urgent HF visits in patients with HF and a left ventricular ejection fraction (LVEF) > 40%. Moreover, in the DELIVER trial, patients with HF with improved LVEF, regardless of care setting (including during hospitalization), were also enrolled [39]. Dapagliflozin led to a statistically significant reduction in the primary composite endpoint of worsening heart failure or cardiovascular death, without a remarkable difference in benefit for patients with an LVEF of ≥60% or less than 60%, or in other subgroups. Furthermore, dapagliflozin resulted in a substantial decrease in the overall number of worsening heart failure events and cardiovascular mortality. The occurrence of adverse effects was comparable to that of the placebo group [39]. Since the AHA, ACCEF, and HFSA’s current guidelines classified SGLT2i as class IIA, level B, for the management of HF with mildly reduced or preserved LVEF [13], the findings reported by the DELIVER study may extend clinical practice guidelines for dapagliflozin usage in HFpEF patients.

Empagliflozin is also a successful approach for HFpEF with LVEF ≤65% since it was able to decrease HF hospitalization and significantly improve Health-Related Quality of Life (HRQOL), as shown in the EMPEROR-Preserved (Empagliflozin Outcome Trial in Patients with Chronic Heart Failure with Preserved Ejection Fraction) trial [40,41,42].

In the SOLOIST-WHF (Effect of Sotagliflozin on Cardiovascular Events in Patients with Type 2 Diabetes Post Worsening Heart Failure) clinical trial, sotagliflozin, a dual SGLT1/SGLT2 antagonist, substantially reduced the incidence of fatal cardiovascular events, hospitalizations, and urgent visits for HF among diabetic patients with worsening HF compared to placebo [18]. The increased incidence of primary endpoint events at 90 days following randomization among placebo-treated patients highlighted that early treatment initiation provides a significant potential to enhance outcomes [18]. The SOLOIST-WHF trial was also designed to assess whether the advantages of SGLT2 inhibition apply to patients with HFpEF, but concise results were difficult to obtain due to the small sample size of this subgroup and early completion date [18]. In major clinical trials, including CREDENCE (Evaluation of the Effects of Canagliflozin on Renal and Cardiovascular Outcomes in Participants with Diabetic Nephropathy) and DAPA-CKD (A Study to Evaluate the Effect of Dapagliflozin on Renal Outcomes and Cardiovascular Mortality in Patients With Chronic Kidney Disease), SGLT2i also showed significant benefits on renal and cardiovascular outcomes in patients with or without type 2 diabetes [43,44]. The ongoing trial EMPA-KIDNEY (The Study of Heart and Kidney Protection with Empagliflozin), with results expected in 2022, assesses the efficacy of empagliflozin in reducing the progression of kidney disease or cardiovascular death in patients with chronic kidney disease [45].

### 2.3. Pharmacological Therapies for HF: Soluble Guanylate Cyclase Activator-Vericiguat

The nitric-oxide-soluble guanylate cyclase (NO-sGC) pathway is altered in decompensated HF due to a reduced NO bioavailability and a shift in the redox state of sGC, which renders it insensitive to NO. Therefore, restoring NO-sGC-cGMP (cyclic guanosine monophosphate) signaling should have the potential to alleviate the HF burden [46,47]. Vericiguat, a new oral sGC stimulator, targets the cGMP pathway by directly activating sGC via a binding site independent of NO. Moreover, by stabilizing NO bound to its site [48], vericiguat administration results in decreased inflammation, fibrosis, and hypertrophy [49].

On the contrary, sGC activators, such as cinaciguat, operate exclusively on abnormal sGC, irrespectively of endogenous NO. In patients with HF, cinaciguat significantly decreased the pulmonary capillary wedge pressure (PCWP) at 8 h, but also increased the incidence of arterial hypotension, which determined the early withdrawal of this trial. Due to its distinctive pharmacokinetic and pharmacodynamic characteristics, vericiguat has a minor impact on arterial blood pressure values when compared with other medications of this class, reducing systolic blood pressure by almost 2 mmHg on average [50,51,52]. In trials comparing vericiguat to placebo, anemia and symptomatic hypotension occurred more often with vericiguat than with placebo [50]. In comparison to other sGC stimulators, such as riociguat, which failed to meet the primary endpoint in phase 2 LEPHT (Riociguat in Patients with Pulmonary Hypertension Associated with Left Ventricular Systolic Dysfunction) interventional clinical trial, modifications to the chemical composition of vericiguat have led to increased pharmacokinetic stability, superior oral bioavailability, and a prolonged half-life, allowing for once-daily oral intake [49,53].

SOCRATES-REDUCED (Phase IIb Safety and Efficacy Study of Four Dose Regimens of Vericiguat in Patients with Heart Failure with Reduced Ejection Fraction Suffering From Worsening Chronic Heart Failure) and VICTORIA (Vericiguat in Participants with Heart Failure with Reduced Ejection Fraction) are the two clinical trials that assessed the safety and effectiveness of vericiguat in HFrEF patients [50,52]. In the SOCRATES-REDUCED trial, changes in NT-proBNP levels after 12 weeks did not vary considerably between the vericiguat and placebo arms, but patients in the vericiguat group had better improvement of the LVEF [52]. The VICTORIA trial proved the effectiveness and safety of vericiguat in patients with HFrEF, with clear benefits in cardiovascular death and HF hospitalization. The patients from VICTORIA were at a higher risk than those enrolled in previous clinical HFrEF trials, as indicated by higher median NT-proBNP values (2816 pg/mL vs. 1608 pg/mL in PARADIGM-HF) as well as patients with NYHA class III or IV symptoms (40% vs. 25% in PARADIGM-HF) [9,50]. On the basis of the results of the VICTORIA study, the 2021 European Society of Cardiology (ESC) HF guidelines recommend that vericiguat may be considered in symptomatic HFrEF patients whose HF has deteriorated even with guideline-directed medical therapy (GDMT) to lower the risk of cardiovascular mortality or HF hospitalization (Class IIb; Evidence Level: B) [14].

In the VITALITY-HFpEF (Outcomes in Vericiguat-treated Patients with HFpEF) trial, the vericiguat alongside standard of care in HFpEF patients did not increase the quality of life assessed by the Kansas City Cardiomyopathy Questionnaire (KCCQ) score [54]. This may be consistent with the hypothesis that NO insufficiency is not the main condition in the development of HFpEF, as opposed to HFrEF [55].

Patients with a recent worsening HF episode and a baseline NT-proBNP value ≥ 8000 pg/mL appear to benefit the most from vericiguat [56]. The newly established benefit and safety of vericiguat in individuals with high-risk HF may encourage the supposition of a quintuple therapy by introducing vericiguat as a novel treatment approach for the treatment of HFrEF, alongside ACEi/ARB/ARNI, beta-blockers, MRA, and SGLTi [57]. In future medical practice, the optimal time, titrating approach, and pharmacological sequencing have yet to be determined.

### 2.4. Pharmacological Therapies for HF: Cardiac Myosin Activation—Omecamtiv Mecarbil

Various drugs that increase cardiovascular outcomes have been identified in patients with HFrEF, but none of them addresses the main drawback of HFrEF, which is impaired systolic function, subsequent decreased cardiac output (CO), and augmented filling pressures [58]. In addition, systolic dysfunction is frequently associated with low levels of arterial blood pressure, which makes it more difficult for patients to tolerate target dosages of GDMT [59]. To maintain optimal CO in HFrEF, present targeted therapies counteract the deleterious implications of hemodynamic and neurohormonal compensatory responses. Beta-adrenergic receptor blockers, ACEI, ARB, ARNi, MRA, hydralazine, and nitrates are evidence-based therapies that improve mortality, whereas ivabradine and digoxin provide benefits for morbidity with no significant improvements in mortality [60]. Those medications that reduce mortality also frequently enhance LVEF and decrease left ventricular end-diastolic volume (LVEDV) and left ventricular end-systolic volume (LVESV), while the majority of drugs that have no effect on mortality fail to improve LVEF [61]. Medications that improve or restore ventricular contractility by targeting underlying mechanisms of the pathophysiology of HFrEF are theoretically promising for both the acute and chronic therapy of HFrEF.

Myosin uses chemical energy to generate force for cardiac myocyte contraction, an activity which is modulated by intracellular calcium levels and regulated by several upstream signaling cascades [62]. Cardiac myosin activators are a novel class of myotropes that improve myocardial function by directly enhancing cardiac sarcomere function [63]. Although several medications have been developed to improve inotropy [64], omecamtiv mecarbil, a cardiac myosin activator, is the first one that enhances systolic function by preferentially enabling the actin–myosin interaction. Thus, omecamtiv mecarbil has the ability to increase the contractile force without any influence on cardiomyocyte calcium handling [62] and without direct impact on vascular, electrophysiological, or neurohormonal processes.

In the COSMIC-HF (Chronic Oral Study of Myosin Activation to Increase Contractility in Heart Failure) interventional clinical trial, 544 patients with HFrEF treated with omecamtiv mecarbil showed an improved left ventricular systolic function, as assessed by a rise in systolic ejection time and EF. Moreover, an improvement in the myocardial strain was also reported, while left ventricular both systolic and diastolic volumes, NT-proBNP, and heart rate (HR) decreased [65,66]. The first trial to demonstrate that selective enhancement of cardiac contractility improves cardiovascular outcomes in patients with HFrEF was GALACTIC-HF (Omecamtiv Mecarbil to Treat Chronic Heart Failure with Reduced Ejection Fraction) [58,67,68]. In this randomized, double-blind, placebo-controlled trial, patients who received omecamtiv mecarbil had a lower incidence of the composite primary outcome of an HF event or death from cardiovascular causes than those in the placebo arm [58]. Patients with an EF below the median (≤28%) showed superior benefits, with a 16% reduction in the primary endpoint [59]. The premise that patients with higher systolic dysfunction would benefit the most from this therapy is plausible and supported by the mechanism of action of omecamtiv mecarbil. Interestingly, omecamtiv mecarbil showed no adverse effect on blood pressure values, heart rate, potassium homeostasis, or renal function. The slight decrease in HR was attributed to sympathetic withdrawal [59]. Although omecamtiv mecarbil treatment showed positive results on primary outcomes, this study failed to demonstrate any improvements in secondary outcomes such as the time to cardiovascular death, change in Kansas City Cardiomyopathy Questionnaire Total Symptom Score (KCCQ-TSS), time to first HF hospitalization, and time to all-cause death [58].

### 2.5. Pharmacological Therapies for HF: Amino Acid Orexigenic Peptide Hormone—Ghrelin

Ghrelin, first discovered in 1999, is a 28-amino acid growth hormone (GH)-releasing peptide, produced mostly by X/A-like cells of the stomach and, to a lesser degree, by the heart and other organs [69,70]. Several research and observational studies suggest that ghrelin presents a myriad of cardioprotective effects through its ability to enhance cardiac contractility; to limit ischemia/reperfusion injury, cardiac cachexia, cardiac hypertrophy, and fibrosis; to lower blood pressure by the inhibition of the sympathetic nervous system; and to ameliorate the prognosis of both myocardial infarction (MI) and HF [71,72,73]. By increasing NO levels and rectifying the endothelin-1/nitric oxide imbalance, ghrelin also has a pivotal role in endothelial function by inducing anti-oxidant, anti-inflammatory, and anti-apoptotic effects [74]. Although several experimental studies have documented the cardiovascular effects of ghrelin, relatively few human clinical trials have been published to date. A low dosage infusion of ghrelin for 60 min in 12 HF patients raised the mean arterial pressure and cardiac and stroke volume index without affecting the heart rate [75]. In another study including 10 patients with congestive HF, intravenous ghrelin administration for three weeks showed substantial improvement of the LVEF and a reduction in LVESV [76]. Furthermore, it improved systolic function and exercise capacity, as measured by a rise in peak workload and peak oxygen consumption during intense activity [76].

These significant and valuable cardiac effects, together with vascular protection, suggest that ghrelin is a promising candidate for the treatment of congestive heart failure and should be further investigated [74]. Synthetic ghrelin that replicates the actions of endogenous ghrelin is widely used for the treatment of metabolic conditions and obesity. However, this peptide may also function as a GH-independent mechanism in cardiomyocytes, a fact that has generally been disregarded by scientists until now [74]. Therefore, additional research is recommended to employ ghrelin as a viable heart failure treatment [77]. All the pharmacological approaches are summarized in Table 1.

Even though real progress has been made in the past decades in the discovery of novel pharmacological treatments for HF, the prevention of premature deaths has only been marginally alleviated. Despite the availability of a plethora of pharmaceutical approaches, proper management of HF is still challenging. Thus, further research, experimental, and clinical studies focusing on the discovery of novel drugs targeting new pathological mechanisms involved in HF are still mandatory.

Recent technological advances have made possible the development of various interventional techniques and device-based approaches for the treatment of cardiovascular diseases. In the following paragraphs, we aimed to summarize the advances made in the development of such procedures and device-based therapies for HF (other than cardiac resynchronization therapy), since these approaches complement and increase the efficiency of the classical drug-based treatments. Moreover, since many of these modern approaches interfere with various well-known described pathological mechanisms in HF, they have a real capability to increase the efficiency of existing medications and improve the prognosis and survival rate. Thus, we consider that among the classical and recently discovered drugs for the treatment of HF, non-pharmacological approaches (other than cardiac resynchronization therapy) must also be discussed.

## 3. Non-Pharmacological Therapies for Heart Failure

### 3.1. Neuromodulatory Approaches

The autonomic nervous system (ANS) plays a critical role in the regulation and homeostasis of the human body, particularly of the cardiovascular system. Since in HF ANS dysregulation has a detrimental effect on cardiac function, improving this pathological alteration by various approaches may represent a pillar in the management of HF [78]. The sympathetic and parasympathetic systems are the two major components of ANS. In the heart, the activation of the parasympathetic nervous system lowers heart rate and decreases contractility, conductance, and myocardial O_2_ consumption, resulting in a reduction in cardiac output during relaxation [79]. Primarily responsible for parasympathetic innervation is the vagus nerve, encompassing all major thoracic organs [80]. Complex interactions between the sympathetic (SNS) and parasympathetic (PNS) nervous systems, as well as regional responses and feedback from the central nervous system, contribute to the modulation of cardiovascular homeostasis [81]. Briefly, excitation of the SNS causes nerve terminals to release norepinephrine (NE), whereas the adrenal glands and medulla release both norepinephrine and epinephrine. These catecholamines bind to adrenergic receptors (ARs), which are further subdivided into subtypes α1, α2, β1, β2, and β3 [82]. In the human heart, β-ARs account for approximately 90% of all ARs, whereas α1-ARs account for almost 10% [83].

HF is characterized by an imbalance of the ANS, which generates a vicious cycle, meaning that the increased sympathetic activity together with reduced vagal activity promote the progression of ventricular remodeling and worsening of heart failure, and likewise, the development of HF further exacerbates the discrepancy between sympathetic and vagal activity [84]. High levels of NE over the long-term enhance myocardial stress due to chronic tachycardia increased afterload and oxygen consumption, thereby worsening ventricular remodeling. Increased catecholamines bind with their own cardiomyocyte β-receptors and stimulate G-protein-coupled receptor kinase upregulation, resulting in the downregulation and desensitization of the β1 receptors at the plasma membrane [81,85,86]. These processes are thought to be protective mechanisms by which the heart preserves itself against severe catecholaminergic toxicity, which commonly induces cyclic adenosine-monophosphate-mediated calcium overload, leading to cardiomyocyte death [81,83,86]. The modulation of the heart PNS is achieved by nicotinic and muscarinic acetylcholine receptors (nAChR and mAChR, respectively) through the neurotransmitter acetylcholine (ACh) [81,87]. Experimental and clinical HF studies have reported that an increased HR, together with a reduced HR variability, is the consequence of PNS dysfunction [88,89].

*Cardiac sympathetic denervation (CSD)* is a surgical antiadrenergic intervention that has significant antiarrhythmic effects, as demonstrated by both preclinical and clinical studies, being effective in severe ventricular arrhythmias [90,91]. CSD decreases automaticity and repolarization heterogeneity and prolongs repolarization. It exerts its effects by interfering with both efferent and afferent neurons [92]. Left-sided sympathetic denervation has been utilized effectively in refractory instances of long QT syndrome, catecholaminergic polymorphic ventricular tachycardia [93], and ventricular arrhythmias in patients with structural heart disease [94,95,96].

*Renal denervation (RDNx)* is a catheter-based procedure used to ablate renal nerves as a solution to ameliorate the pathophysiology of HF by lowering the activity of the sympathetic nervous system. In both HF experimental and clinical studies, RDNx is able to induce antihypertensive effects but also improve adverse cardiac remodeling [97,98,99,100]. The REACH-pilot study was the first to evaluate the value of RDNx in HF symptomatic patients. In the study, RDNx was related to improvements in both symptoms and exercise ability. There was neither a substantial fall in blood pressure nor a decline in renal function, and some patients were able to limit their usage of diuretics [101]. In the clinical studies conducted so far, RDNx seems to be safe and well tolerated in patients with HFrEF by improving HF symptoms and modestly lowering systolic and diastolic blood pressure without worsening renal function [102]. Further insights into the mechanisms by which RDNx improves the physiopathology of HF are required. In this regard, clinical trials with control arms such as RE-ADAPT-HF (A Prospective, Multicenter, Randomized, Blinded, Sham-controlled, Feasibility Study of Renal Denervation in Patients with Chronic Heart Failure) and UNLOAD-HFpEF (Renal Denervation to Treat Heart Failure with Preserved Ejection Fraction) are ongoing, with the results expected in the next years.

*Vagus nerve stimulation (VNS)*. During an inflammatory response, the vagus nerve acts as an afferent and efferent pathway between the brain and peripheral organs, including the heart [103]. In the presence of proinflammatory cytokines in the periphery, the sensory afferents of the vagus nerve are activated and transmit the signal to the brain. This signal induces the release of acetylcholine from the vagus nerve efferents into the reticuloendothelial system, which limits inflammation by reducing the synthesis and release of proinflammatory cytokines [104]. Thus, it is comprehensible that VNS might reduce the proinflammatory state, which is already recognized as a critical pathogenic mechanism in HF, particularly in HFpEF, since it is associated with promoting cardiac remodeling [104]. Schwartz and colleagues were the first to describe the efficacy of long-term VNS in patients with heart failure. They reported an improvement in functional status, quality of life, and left ventricular volume in HFrEF after vagus nerve stimulation [105]. However, larger clinical studies of VNS in patients with HFrEF, such as NECTAR-HF (Neural Cardiac Therapy for Heart Failure Study), INOVATE-HF (Increase of Vagal Tone in Chronic Heart Failure), and ANTHEM-HF (Autonomic Neural Regulation Therapy to Enhance Myocardial Function in Heart Failure) have not reliably reproduced the advantages so far [106,107,108]. The inconsistency may be the result of extensive variation in stimulation settings, targets, and systems. ANTHEM HFrEF (Autonomic Regulation Therapy to Enhance Myocardial Function and Reduce Progression of Heart Failure with Reduced Ejection Fraction) is an adaptive, open-label, randomized, controlled study that is now enrolling and is expected to provide more insights regarding the efficiency of VNS on HF outcomes [109].

*Tragus nerve stimulation*. One of the main drawbacks of vagus stimulation is the invasive nature of this therapy, which is accompanied by surgical risks and low patient tolerance [110]. Low-level tragus stimulation (LLTS) is a non-invasive transcutaneous approach that may influence autonomic function by stimulating the auricular branch of the vagus nerve (ABVN) [111]. Currently, ideal LLTS parameters are unclear. In both preclinical and clinical research, LLTS parameters have been empirically determined. In a rat model of heart failure with HFpEF, LLTS lowered both systolic and diastolic blood pressure. Furthermore, left ventricular hypertrophy, circumferential strain, and diastolic function were improved. It has also reduced inflammatory cell infiltration and fibrosis within the ventricle and induced downregulation of pro-inflammatory and pro-fibrotic genes [112]. Human trials of LLTS in HF patients are very limited. In a prospective, randomized, double-blind, 2 × 2 cross-over trial, 1 h of LLTS improved the longitudinal mechanics of the left ventricle and the heart rate variability (HRV) in patients with HFrEF [113]. In a pilot, randomized, sham-controlled research including patients with HFrEF, 1 h of LLTS improved microcirculation as assessed by flow-mediated vasodilatation [114].

*Cardiac contractility modulation (CCM)* is a novel approach that employs non-excitatory electrical impulses to the interventricular septum during the absolute refractory period [115]. Implantation is similar to a conventional transvenous pacemaker device, except two right ventricular leads are used. Mechanistic research has shown an increase in left ventricular contractility and positive global effects on reverse remodeling, mostly as a result of calcium handling improvements by phosphorylation of phospholamban and upregulation of SERCA-2A [116]. Increases in functional ability and quality of life have been shown in clinical trial data, but long-term outcome data are limited [117]. After two pilot studies to validate the safety and feasibility of CCM in patients with HFrEF [118], FIX-HF-3 was the first observational trial to evaluate the clinical efficacy of CCM treatment in 25 patients [119]. At a 2-month follow-up, improvements were found in LVEF, 6 min walk distance (6MWD), NYHA functional class, and quality of life in HFrEF NYHA III patients [120]. This was accompanied by the first randomized, double-blind crossover trial (FIX-CHF-4), which included patients with severe HFrEF, defined as LVEF < 35%, NYHA class II-III, and a narrow QRS duration. Those who were medically optimized were compared to those who received additional CCM therapy, with measures taken after 12 weeks in each group. Peak VO2 as assessed by cardiopulmonary exercise testing (CPEX) improved similarly in both groups (0.4 mL/kg/min), which strongly indicated a placebo effect. After the 6-month treatment period, however, only those with CCM showed a consistent improvement linked with QoL indicators. In a recent randomized controlled study, FIX-HF-5C (Evaluation of the Safety and Effectiveness of the OPTIMIZER System in Subjects with Heart Failure), which enrolled patients with NYHA class III or IV symptoms, QRS length of 130 ms, and LVEF between 25 and 45%, patients in the CCM arm had statistically significant improvements in NYHA class, 6MWD, and quality of life, as well as a composite reduction in HFrEF hospitalization and cardiovascular mortality. Moreover, a subgroup analysis of the FIX-HF-5 study revealed more substantial treatment advantages, such as that CCM therapy may provide additional benefits in patients with a relatively moderate LVEF decline [121,122].

*Baroreceptor activation therapy (BAT).* The carotid body and sinus are innervated by both PNS (via the vagus and glossopharyngeal fibers) and SNS (via cervical sympathetic ganglia). Electrical stimulation of carotid sinus baroreceptors generates afferent signals to the dorsal medulla, resulting in SNS reduction and enhanced vagal tone, which reduces blood pressure and heart rate [123,124]. In HF patients, these responses associated with the baroreceptor pathway are partially blunted due to carotid body alterations, leading to baroreflex dysfunction and subsequent SNS overactivation [123]. Baroreceptor sensitivity impairments in heart failure are related to higher death rates [125]. In preclinical studies, BAT was shown to diminish sympathetic tone and increase parasympathetic signaling, thus enhancing the autonomic input to the heart [126]. The BeAT-HF (Baroreflex Activation Therapy for Heart Failure) clinical trial demonstrated that BAT is safe in HFrEF patients and significantly improves patient-centered symptomatic endpoints of the QOL score, exercise capacity, and functional status [127]. Moreover, a considerable improvement in NT-proBNP levels was achieved with BAT, despite a disproportionate rise in the number of drugs in the control group [127].

*Endovascular Ablation of the Right Greater Splanchnic Nerve (GSN).* Exertional dyspnea, decreased aerobic capacity, and higher mortality are all linked to elevated intracardiac filling pressures at rest and during exercise in HF patients with both reduced and preserved ejection fraction [128,129,130]. As a result, several cardiovascular therapies aim to lower intracardiac filling pressures in these patients to enhance exertional capacity and QOL and improve cardiovascular morbidity [131].

Reduced inotropic and chronotropic reserves, as well as impaired relaxation, all contribute to higher filling pressures at rest and during activity. The vascular system is also involved in this process by reducing pulmonary arterial compliance and increasing pulmonary arterial resistance. Excessive blood volume distribution from extrathoracic compartments into the thorax is a major factor in high filling pressures in HF patients [131,132]. Splanchnic vasoconstriction mediated by the SNS causes rapid blood shifts from the splanchnic compartment to the heart and lungs, which is a typical physiological adaptative response mechanism during exercise. These rapid blood volume shifts from the splanchnic to central vasculature, however, cause an exaggerated rise in heart filling pressures in patients with HF, increasing exercise intolerance and possibly leading to HF decompensation [133,134].

Splanchnic nerve activity modulation has thereby been developed as a possible treatment approach in HF patients to reduce volume redistribution and improve symptoms and outcomes. Recent research has explored the impact of temporarily and permanently blocking the GSN over the HF spectrum. Splanchnic nerve modulation has been proven beneficial for both acute decompensated (ADHF) and chronic heart failure (CHF), according to the Splanchnic Nerve Anesthesia in Heart Failure and Abdominal Nerve Blockade in Chronic Heart Failure trials. In 11 ADHF patients with advanced HFrEF who underwent short-term blockade of the greater splanchnic nerve via anesthetic agents, there was a significant decrease in PCWP and an increase in the cardiac index [135]. Comparable outcomes were yielded from a study including 18 CHF patients who underwent the same procedure [136]. In HFpEF patients, permanent ablation of the right greater splanchnic nerve led to a decrease in intracardiac filling pressures during exercise as soon as 24 h following the intervention [137]. The Surgical Resection of the Greater Splanchnic Nerve in Subjects Having Heart Failure with Preserved Ejection Fraction two-center study has shown a substantial decrease in PCWP at the 3-month follow-up and a considerable 12-month improvement in NYHA class and QOL [132]. To ablate the right-sided GSN, a novel, endovascular, transvenous, minimally invasive procedure (splanchnic ablation for volume management-SAVM) was designed, and it has been proven to be helpful in a small, single-center open-label pilot trial [138].

REBALANCE-HF (Endovascular Ablation of the Right Greater Splanchnic Nerve in Subjects Having HFpEF) is an ongoing, multicenter, randomized, sham-controlled trial whose objective is to assess the safety of unilateral ablation of the right greater splanchnic nerve and its effectiveness in improving hemodynamics, quality of life, and exercise tolerance in patients with HFpEF [139]. The preliminary results from this trial show that GSN ablation is efficient in reducing PCWP during exercise, with improving the symptoms, but without a significant change in exercise capacity. The decrease in PCWP is substantial and it is consistent with previous results suggesting that abnormalities in venous capacitance play a significant role in the development of hemodynamic perturbations in HFpEF during exercise [140]. These findings show for the first time that endovascular GSN ablation can be used to treat HFpEF. All neuromodulatory approaches are summarized in Table 2.

### 3.2. Respiratory Disorders Implicated in Heart Failure

Sleep-disordered breathing, a widespread condition affecting both the circulatory and respiratory systems, is one element now recognized as contributing to the increased morbidity and mortality in HF. Two primary sleep apnea syndromes have been described: obstructive sleep apnea syndrome (OSA) and central sleep apnea syndrome (CSA) [109].

*Stimulation of Phrenic Nerve.* This procedure involves inserting an electrode into a brachiocephalic or pericardiophrenic vein to detect the diaphragm’s contractions throughout breathing and activate the diaphragmatic nerve during apnea, with the purpose to preserve fairly constant pO_2_ and pCO_2_ levels and avoid SNS and RAAS overactivation [141,142]. In the pivotal trial of the remedé system (Respicardia Inc., Minnetonka, MN, USA) involving 151 patients, the stimulation of the phrenic nerve showed a substantial decrease in the apnea–hypopnea index (AHI), central apnea index, arousal index, oxygen desaturation 4% index, percentage of sleep with rapid eye movement, and sleepiness (Epworth Sleepiness Scale (ESS)) [143]. A 5-year follow-up investigation confirmed these findings [144]. According to Costanzo et al., patients who received phrenic nerve stimulation displayed an increase in the QOL and LVEF without a substantial change in end-systolic and end-diastolic volumes [141]. Large-scale clinical studies are necessary to determine the impact of phrenic nerve stimulation on mortality in individuals with HF and CSA syndrome [145].

*Synchronized Diaphragmatic Therapy.* Increased intrathoracic pressure exerts persistent stress on the heart muscle and may exacerbate heart failure (HF). The respiratory muscles have a substantial effect on intrathoracic pressure, and, thus, the implantation of a device coupled to an electrode that detects the heartbeat that activates the diaphragm was developed [145]. In the Stimulation of the Diaphragm in Patients with Severe Heart Failure Following Heart Surgery randomized trial including 33 subjects, an improvement in LVEF and HF symptoms, and an elevation in maximal power and oxygen consumption during exercise testing was noticed, with no considerable improvement in the six MWT, nor the BNP levels [146]. At the 1-year follow-up of the non-randomized VisOne Heart Failure trial, improvements in LVEF, QOL, and 6MWT were reported [147]. Although both trials included a limited number of patients, because of the encouraging outcomes, it might be beneficial to conduct additional research on a larger scale.

### 3.3. Devices for Decongestion in HF

Although loop diuretics continue to represent the backbone of decongestive treatment in HF, the occurrence of drug resistance, particularly with prolonged usage, poses a therapeutic issue that requires the development of novel approaches [145].

TARGET-1 and TARGET-2 (A Study to Evaluate the Treatment of Patients with Acute Decompensated Heart Failure (ADHF) Using an Automated Fluid Management System) trials evaluated the safety and effectiveness of controlled decongestion using the Reprieve system, which is intended to detect urine output and administer a specific amount of substitute solution to reach the predefined fluid balance. In both trials, patients experienced an increase in urine output, a decrease in body weight, and a drop in central venous pressure (CVP), while the SBP remained constant and without renal dysfunction [148]. The first human trials of the Doraya catheter, a device designed to transiently lower renal venous pressure by generating a manageable gradient in the inferior vena cava just under the renal veins, have shown encouraging results [149]. The Doraya catheter appears to represent an innovative idea for the management of AHF patients with poor diuretic response, whereas the Reprieve device is intended for AHF patients responsive to diuretics [145].

The VENUS-HF (VENUS-Heart Failure Early Feasibility Study) regarding the preCARDIA system, a device implanted into the superior vena cava to induce transient blockage, resulting in a reduction in right ventricular preload, revealed a reduction in right atrial pressure and PCWP [150]. The WhiteSwell device, intended to produce a low-pressure zone in the outflow of the thoracic duct into the venous system, has been studied in both animal and human studies. In the animal experiment, WhiteSwell not only prevented the collection of more fluid but also stimulated its discharge [151]. The orthopnea and oedema improved when the device was applied to humans. The Aquapass system is a wearable device designed to raise the skin temperature of the lower body without affecting the body’s core temperature. Increasing sweat rate in HF patients appears to be a reasonable option for decongestive treatment; nevertheless, further research is required to determine the method’s particular usefulness and effectiveness [152].

### 3.4. Ongoing Trials for Non-Pharmacological Therapies for HF

In the ALLEVIATE-HF-1 (NCT04583527), ALLEVIATE-HF-2 (NCT04838353), and ALLEVIATE-HFrEF (NCT05133089) studies, patients with HFpEF, HFmEF, and HFrEF will be enrolled for treatment through a no-implant interatrial shunt, using clinical, echocardiographic, and invasive hemodynamic data. The transcatheter system is designed to lower left atrial pressure by developing a therapeutic interatrial shunt, without the need for a permanent cardiac implant or open-heart surgery.

RELIEVE-HF (Reducing Lung Congestion Symptoms in Advanced Heart Failure- NCT03499236), a randomized clinical trial, is evaluating the impact of the V-Wave Ventura Interatrial Shunt System on heart failure patients, including the ability to lower hospitalizations and improve symptoms, exercise capacity, and quality of life. This small, hourglass-shaped device facilitates blood to flow from the left to the right atrium, lowering the pressure on the left side during physical activity.

**Table 2:** Neuromodulatory approaches in HF.

Neuromodulatory Approaches	Mechanisms of Action	Clinical Trial/Study	Main Findings	Limitations
**Cardiac sympathetic denervation**	surgical antiadrenergic denervation	Vaseghi et al. Schwartz et al.	→antiarrhythmic effects; →improvements in HR variability and autonomic nervous system [90,91,92,93,94,95,96]	→limited data exist on the benefits of sympathetic denervation in HF patients.
**Renal denervation**	frequency-based catheter renal nerve ablation	REACH pilot study	→improvements in both symptoms and exercise ability [101]	→the RDT-PEF (Renal Denervation in Heart Failure with Preserved Ejection Fraction) trial was prematurely disrupted due to enrollment challenges, leaving it underpowered to determine whether RDN positively affected QOL, exercise function, biomarkers, and left heart remodeling in HFpEF patients [153].
		RE-ADAPT-HF UNLOAD-HFpEF	→enrolling →enrolling	→future randomized, blinded, sham-controlled clinical studies are necessary to establish the impact of RDN on the morbidity of HFrEF and HFpEF patients.
**Vagus nerve stimulation (VNS)**	electrical stimulation of the vagus nerve	Schwartz et al.	→improvement in functional status, quality of life (QoL), and left ventricular volume in HFrEF [105]	
		NECTAR-HF (NCT01385176)	→favorable long-term safety profile; failed to show that VNS improved clinic outcomes versus OMT [106]	→VNS has a considerable favorable effect on the functional state of the patient, but with no effect on the prognosis [107].
		INOVATE-HF (NCT01303718)	→quality of life, NYHA class, and 6 min walking distance were favorably affected by vagus nerve stimulation; failed to show that VNS improved clinic outcomes versus OMT [107]	→the lack of a control group in the ANTHEM-HF trial is a considerable limitation; to avoid the placebo effect and validate the procedure’s safety, a randomised, controlled clinical trial is required [108].
		ANTHEM-HF (NCT01823887)	→chronic open-loop left- or right-side VNS is feasible and well tolerated in HFrEF patients [108]	→no significant echocardiographic improvements nor reduction levels of NTpro BNP have been documented in any study [107].
		ANTHEM-HFrEF (NCT03425422)	→enrolling; test the impact of Vitaria system on cardiovascular mortality and HF hospitalization in patients with HF and reduced EF (HFrEF) [109]	
**Tragus nerve stimulation**	non-invasive transcutaneous approach to VNS that stimulates the auricular branch of the vagus nerve	Zhou et al.	→lowered both systolic and diastolic blood pressure; →left ventricular hypertrophy, circumferential strain, and diastolic function; →reduced inflammatory cell infiltration and fibrosis within the ventricle and induced downregulation of pro-inflammatory and pro-fibrotic genes [112]	→previous research has a number of limitations, including the absence of a well-controlled placebo group and longitudinal data and the limited sample populations; the optimal stimulation settings have yet to be established. →longitudinal data are required to assess the long-term impact of LLTS.
		Tran et al.	→ improved the longitudinal mechanics of the left ventricle and the heart rate variability (HRV) in patients with HFrEF [113]	→moreover, there is no validated biomarker for measuring the efficacy of LLTS [154].
		Dasari et al.	→ improved microcirculation [114]	
**Cardiac contractility modulation (CCM)**	myocardial non-excitatory electrical impulses delivered during the absolute refractory period that increases left ventricular contractility as a result of calcium handling improvements by phosphorylation of phospholamban and upregulation of SERCA-2A	FIX-HF-3	→improvements in LVEF, 6 min walk distance (6MWD), NYHA functional class, and quality of life in HFrEF NYHA III patients [119,120]	→the impact of CCM on parameters such as left ventricular diastolic volumes has not been investigated systematically.
		FIX-CHF-4	→consistent improvement linked with QoL indicators at 6 months of therapy in HFrEF patients who received CCM [121]	→CCM may only be effective when administered to viable, non-necrotic myocardium; however, this has not been fully investigated in preclinical or clinical research.
		FIX-HF-5 (NCT00112125)	→subgroup analysis revealed improvements in ventilatory anaerobic threshold were observed in patients with ejection fraction ranging from 25% to 45% [121,122]	→likewise, the advantages of CCM in CRT “non-responder” patients are inadequately documented. →in the study conducted by Kuschyk et al., there was an increased number of adverse outcomes, including two fatalities.
		FIX-HF-5C (NCT01381172)	→statistically significant improvements in NYHA class, 6MWD, QoL, a composite reduction in hospitalization, and cardiovascular mortality [122]	→prospective trial results are inadequate, and it is essential that this disparity be settled prior to expanding usage in populations with medically optimal adjusted HFrEF, narrow QRS duration, and persistent symptoms [116].
**Baroreceptor activation therapy (BAT)**	electrical stimulation of carotid sinus baroreceptors lowers SNS activity and increases parasympathetic tone	BeAT-HF (NCT02627196)	→BAT is safe and effective; →BAT significantly improved QoL and 6MWD, and reduced NT-proBNP levels [127]	→BAT requires larger-scale studies with extended follow-up periods, a wider cohort of patients, and defined outcomes, including mortality risks, before this procedure can be included in HF clinical practice [155].
**Splanchnic nerve modulation (SNM)**	modulation of splanchnic nerve activity reduces cardiac filling pressures	REBALANCE-HF (NCT04592445)	→the preliminary results from this ongoing trial show that GSN ablation is efficient in reducing PCWP during exercise, with improving the symptoms but without a significant change in exercise capacity [140]	→the safety and effectiveness of SNM in the management of HF must be explored more extensively; the latest scientific studies are centered on limited patient groups with minimal follow-up; the aforementioned proof-of-concept clinical trials lacked a control group [132].

It is a certainty now that the technological advances from the last years have paved the way for the development of non-pharmacological approaches that may efficiently complement classical HF therapy. Although further experimental studies are required to elucidate the underlying mechanisms through which many of these therapies act, the promising and encouraging results reported to date compel us to extend the HF treatment beyond the classical view.

## 4. Future Perspectives of HF Management—Artificial Intelligence

A late-breaking discovery presented at the European Society of Cardiology’s (ESC) Heart Failure 2022 congress was a voice analysis software that can be used by heart failure patients at home that can detect fluid in the lungs in up to 80% of cases, three weeks prior to an unexpected hospitalization or escalation in outpatient medication therapy [156]. As cardiovascular illnesses evolve, advances in therapeutic and diagnostic approaches are required, and artificial intelligence (AI) is now being rapidly integrated into the field of cardiovascular medicine. By analyzing colossal databases more effectively than the human brain, AI has the potential to improve medical diagnosis, treatment, risk prediction, clinical care, and drug development [157]. For healthcare providers, AI has the potential to reduce the risk of adverse events, patient waiting times, and per capita expenditures while boosting accessibility, productivity, and overall patient experience [158]. AI also has the potential to reduce workloads and margin of error for physicians, as well as to improve patient–doctor interactions and therapeutic decision making [159,160]. For patients, AI can improve their health and well-being by increasing their knowledge, shared decision making, and self-efficacy in disease management [160].

For accurate quantitative and qualitative evaluation of heart failure, AI has been incorporated into different cardiac imaging techniques, such as echocardiography, cardiac magnetic resonance imaging, and cardiac computed tomography. Machine learning algorithms have been found to deliver a near-instantaneous echocardiography evaluation. Knackstedt et al. showed that the LVEF and longitudinal strain could be determined in less than 8 s [161]. This quick and precise evaluation might also have applications outside the cardiology department, such as in the emergency room, where point-of-care ultrasound scans are becoming more popular [162]. AI has been also shown to be of crucial importance in cardiac magnetic resonance imaging, especially for ventricular segmentation [163]. Laser et al. compared knowledge-based reconstruction of right ventricular volumes to the gold standard of direct cardiac MRI, finding that knowledge-based reconstruction offers outstanding accuracy for right ventricular 3D volumetry [164]. AI-assisted 3D visualization and cardiac image reconstruction can aid in the identification of a range of disorders [165,166]. Similarly, completely automated AI systems have provided a considerably more accurate calculation of left ventricular mass, papillary muscle identification, common carotid artery, and descending aorta measurements [167,168]. AI has been increasingly used for cardiac computed tomography, particularly for the assessment of coronary artery calcification scoring and risk stratification of future events [169]. Interestingly, AI uses various risk calculation scoring systems to estimate cardiovascular mortality and predictive models to predict the risk of future hospitalization so that proper monitoring and control may be carried out to avoid such harmful results [140,141,142,143,144,145,146,147,148,149,150,151,152,153,154,155,156,157,158,159,160,161,162,163,164,165,166,167,168,169,170,171,172,173,174,175].

Current HF healthcare services are insufficient to satisfy the demands of an ageing population with rising comorbidities and disease complexity, as well as the disparity in medical care distribution between rural and urban areas. As a result of these factors, an urgent need to develop alternative healthcare treatments has arisen. eHealth apps have the ability to relieve a large amount of the strain on healthcare services while also improving patient care. The PASSION-HF (Patient Self-Care using eHealth in Chronic Heart Failure) project intends to create a virtual doctor, a digital decision support system that offers options based on current clinical standards. Patient independence is enhanced by providing tailored HF management 24 h a day, 7 days a week. In addition, the program establishes processes and decision points at which medical experts must be involved [176].

Although AI has the potential to solve many of the fundamental challenges faced by the current HF pandemic, it is still a fast-growing field, and therefore some caution is advised. Transparency in data quality, population representativeness, and performance evaluation will be critical. Clinicians, patients, caregivers, and IT professionals should all be included in discussions about legal, technological, and regulatory problems, with ethics and equity being prioritized [177].

## 5. Conclusions

Heart failure is becoming an irrefutably significant disease entity as the population ages. Thus, various mechanisms contributing to the development and progression of HF have been discovered and targeted with novel medications and non-pharmacological approaches throughout the last three decades. This has improved the clinical outcome of millions of people worldwide with HF in terms of mortality, quality of life, and survival. Researchers are aiming to identify subgroups in which specific drugs and/or devices may be most successful, innovative methods for enhanced diagnosis and prediction of prognosis in HF patients, and novel tools for treating HF. New therapies will hopefully bring more benefits and extend these results to the treatment of HFpEF also, as well as other causes and phenotypes of HF.

## Figures and Tables

**Table 1:** Pharmacological therapies in HF.

Drug Class		Clinical Trial/Study	Main Findings	Ongoing Trials
**Dual angiotensin receptor and neprilysin inhibitors (ARNI)**	Omapatrilat	OVERTURE	→not superior to an angiotensin-converting enzyme (ACE) inhibitor alone in lowering the rate of heart failure (HF) hospitalization or mortality risk [7]	
		OCTAVE	→omapatrilat group was more likely to reach blood pressure target; →increased incidence of angioedema [8]	
	Sacubitril/valsartan	PARADIGM-HF (NCT01035255)	→superior to enalapril in reducing the risks of death and heart failure hospitalization (HHF); →decreased the symptoms and physical limitations of HF; →lower incidence of renal function impairment, hyperpotassemia in sacubitril/valsartan group [9]	1. PARAGLIDE-HF (NCT03988634) will assess the effects of sacubitril/valsartan vs. valsartan monotherapy on NT-proBNP levels, clinical outcomes, safety, and tolerability in HFpEF patients admitted for acute decompensated HF. 2. NCT04587947 will assess the effect of sacubitril/valsartan on the autonomic cardiac nerve system by monitoring HRV in HF patients. 3. TurkuPET (NCT03300427) will assess the effects of six weeks of sacubitril/valsartan versus valsartan on cardiac oxygen consumption and cardiac work efficiency in patients with NYHA class II and III HFrEF.
		PIONEER-HF (NCT02554890)	→in acute decompensated heart failure with reduced ejection fraction (HFrEF), a greater reduction in the N-terminal pro B-type natriuretic peptide (NT-proBNP) concentration was obtained with sacubitril–valsartan than with enalapril [10]	4. NCT04688294 will assess the effects of sacubitril/valsartan in the treatment of congestive HF patients, as well as the drug’s adverse effects by monitoring renal function and serum electrolytes. 5. ARNICFH (NCT05089539) will assess the effects of ARNI on cardiac fibrosis in HFpEF patients. 6. NCT03928158 will assess the effects of sacubitril/valsartan vs. valsartan treatments in patients with advanced LV hypertrophy and HFpEF. 7. PARABLE (NCT04687111) will assess the hypothesis that sacubitril/valsartan might improve left atrial structure and function as well as left ventricular structure and function in asymptomatic HFpEF patients. 8. ENVAD-HF (NCT04103554) will assess sacubitril/valsartan in advanced HF and left ventricular assist device recipients.
		PARAGON-HF (NCT01920711)	→no significant benefit in patients with HF and preserved ejection fraction (HFpEF) regarding total hospitalizations for HF and death from cardiovascular causes [12]	
**Sodium-glucose co-transporter-2 inhibitors (SGLT2i)**	Canagliflozin	CANVAS (NCT01032629 and CANVAS-R (NCT01989754)	→in patients with type 2 diabetes (T2D) and an elevated risk of cardiovascular disease, canagliflozin treatment was associated with a lower risk of cardiovascular events; → possible benefit of canagliflozin in preventing the progression of albuminuria [16]	1. NCT05364190 will assess the efficacy and safety of the early initiation of canagliflozin treatment in hospitalized heart failure patients with volume overload (warm-wet) who require the use of an I.V loop diuretic during the hospitalization period.
		CREDENCE (NCT02065791)	→in patients with T2D and kidney disease, canagliflozin treatment showed a lower risk of kidney failure and cardiovascular events [16]	
	Dapagliflozin	DECLARE-TIMI58 (NCT01730534)	→in patients with T2D at risk for atherosclerotic cardiovascular disease, dapagliflozin treatment was associated with a lower rate of cardiovascular death or HHF [17]	1. DAPA-RESPONSE-AHF (NCT05406505) will assess the effect of dapagliflozin in patients with acute heart failure.
		DAPA-HF (NCT03036124)	→in patients with HF, dapagliflozin was superior to placebo at preventing cardiovascular deaths and heart failure events, irrespective of the presence or absence of diabetes [19]	NCT05346653 will assess the effects of SGLT2i in acute decompensated heart failure.
		PRESERVED-HF (NCT03030235)	→12 weeks of dapagliflozin treatment significantly improved symptoms, physical limitations, and exercise function in HF with preserved ejection fraction (HFpEF) patients [38]	NCT05278962 will assess the outcomes of SGLT2i in HF patients with left ventricular assist devices.
		DELIVER (NCT03619213)	→trial completed with results regarding the efficacy and safety of dapagliflozin in HFpEF patients available later in 2022 [39]	ICARD (NCT05420285) will assess the cardiometabolic mechanistic effects on the myocardium of dapagliflozin in HFrEF patients.
	Empagliflozin	EMPA-REG OUTCOME (NCT01131676)	→superior to placebo in reducing cardiovascular events, including cardiovascular death, all-cause mortality, and HHF [15]	1. DRIP-AHF-1 (NCT05305495) will assess the effect of empagliflozin in acute heart failure. 2. NCT05139472 will assess the impact of empagliflozin on functional capacity in HFpEF.
		EMPEROR-Reduced (NCT03057977)	→superior to placebo in improving HF outcomes (cardiovascular death or HHF) [32]	
		EMPEROR-Preserved (NCT03057951)	→reduced the combined risk of cardiovascular death or HHF in HFpEF patients [40]	
		EMPA-CKD (NCT03594110)	→ongoing trial; it assesses the effect of empagliflozin on kidney disease progression or cardiovascular death versus placebo	
	Sotagliflozin	SOLOIST-WHF (NCT03521934)	→a significantly lower total number of deaths from cardiovascular causes and hospitalizations and urgent visits for HF than placebo [18]	
**Soluble guanylate cyclase activator (sGC)**	Vericiguat	SOCRATES-REDUCED (NCT01951625)	→in patients with worsening chronic HF and reduced left ventricular ejection fraction (LVEF), no statistically significant effects on NT-proBNP levels at 12 weeks was observed in the vericiguat group [52]	1. VICTOR (NCT05093933) will assess the efficacy and safety of vericiguat in HFrEF patients, specifically those with symptomatic chronic HfrEF who have not had a recent hospitalization for heart failure or need for outpatient intravenous (IV) diuretics.
		VICTORIA (NCT02861534)	→a lower incidence of death from cardiovascular causes or HHF in patients receiving vericiguat [50]	
		VITALITY-HfpEF (NCT03547583)	→no improvement in the quality of life (QoL) at 24 weeks in HfpEF patients receiving vericiguat [54]	
**Cardiac myosin activators**	Omecamtiv mecarbil	COSMIC-HF (NCT01786512)	→improvement of systolic ejection time, stroke volume, left ventricular end-diastolic diameter, heart rate, and NT-proBNP in the pharmacokinetic-titration group [65]	
		GALACTIC-HF (NCT02929329)	→a lower incidence of the primary composite of an HF event or death from cardiovascular causes in the omecamtiv mecarbil group than placebo [59]	

## Data Availability

Not applicable.
